# UVC irradiation suppresses platelet-derived growth factor-BB-induced migration in human pancreatic cancer cells

**DOI:** 10.3892/or.2011.1612

**Published:** 2011-12-23

**Authors:** JUNJI KAWAGUCHI, SEIJI ADACHI, ICHIRO YASUDA, TAKAHIRO YAMAUCHI, TAKASHI YOSHIOKA, MASAHIKO ITANI, OSAMU KOZAWA, HISATAKA MORIWAKI

**Affiliations:** 1 Department of Gastroenterology, Gifu University Graduate School of Medicine, Gifu 501-1194, Japan; 2 Department of Pharmacology, Gifu University Graduate School of Medicine, Gifu 501-1194, Japan

**Keywords:** UVC, platelet-derived growth factor-BB, cell migration, pancreatic cancer, Akt

## Abstract

We have recently reported that short wavelength ultraviolet-C (UVC) irradiation inhibits cell growth and induces apoptosis in human pancreatic cancer cells. In this study, we investigated the effect of UVC on platelet-derived growth factor (PDGF)-BB-induced migration in pancreatic cancer cells, AsPC1 and BxPC3. In cell migration assays using a Boyden chamber Transwell, PDGF-BB exerted a maximum effect on migration of these cells at a dose of 70 ng/ml after 36 h of treatment. PDGF-BB also caused phosphorylation of p44/p42 mitogen-activated protein kinase (MAPK), stress-activated protein kinase/c-Jun-N-terminal kinase (SAPK/JNK) and Akt, but not of p38 MAPK in these cells. Pretreatment of these cells with UVC at a dose over 10 J markedly suppressed PDGF-BB-induced migration. Since UVC significantly inhibited PDGF-BB-induced phosphorylation of Akt, and subsequent glycogen synthase kinase (GSK) 3β, but not p44/p42 MAPK and SAPK/JNK, it is likely that UVC inhibits PDGF-BB-induced migration by suppressing the Akt-GSK3β pathway in pancreatic cancer cells. Taken together with our previous findings, UVC could be a useful tool for the treatment of patients with pancreatic cancer.

## Introduction

Pancreatic cancer is a common malignancy, ranking the thirteenth in the incidence, and the eighth as the cause of cancer-related deaths worldwide (1). Because of the difficulty in the early diagnosis of pancreatic cancer, most patients with this malignancy have already reached an advanced stage when the first symptoms appear. The standard treatment for advanced pancreatic cancer is chemotherapy. Although gemcitabine is the first line drug for pancreatic cancer, the median survival of patients treated with gemcitabine is not satisfactory. Therefore, researchers expanded their interests to the development of new treatments for inoperable pancreatic cancer.

Cell invasion into adjacent tissues is a major prognostic factor for advanced pancreatic cancer patients. Abnormal cell migration leads to pathological states such as invasion and metastasis of cancer. The cytoskeleton, which is composed of actin filaments and a network of microtubules, controls cell motility (2). Actin stress fibers generate contractile forces by pulling against focal adhesions to induce retraction of the rear cell membrane, suggesting that stress fibers are important for cell migration (3). Cytoskeletal proteins such as vinculin, actinin, and several non-receptor protein tyrosine kinases, including members of the Src family and the focal adhesion kinase (FAK), are involved in the organization of focal adhesion complexes (4,5).

The platelet-derived growth factor (PDGF) family is comprised of four different polypeptide chains encoded by different genes, which have been identified as PDGF-A, PDGF-B, and the recently discovered PDGF-C and PDGF-D. So far, four homodimers (PDGF-AA, PDGF-BB, PDGF-CC and PDGF-DD) and one heterodimer (PDGF-AB) have been described (6). PDGF and its receptor are known to have a role in the pathogenesis, invasion, and distant metastasis of human solid tumors and their expressions are correlated with poor prognosis (7,8).

Ultraviolet (UV) radiation from sunlight is sorted by wavelength regions: long-wavelength UVA (320–400 nm), medium-wavelength UVB (280–320 nm) and short-wavelength UVC (200–280 nm). UVA and UVB are recognized as the major carcinogenic components of sunlight (9). On the other hand, UVC does not actually reach the earth surface since it is filtered out by the atmosphere, it is generally used for studying DNA damage and cellular DNA repair process, and is commonly applied for equipments such as water sterilization. Recently, the application of UVC for the treatment of human cancer has been suggested (10,11). Similarly, we recently reported that UVC irradiation induces cell growth via downregulation of epidermal growth factor receptor (EGFR) (12) and also induces apoptosis (13) in human pancreatic cancer cells. Moreover, our recent study showed that UVC induced evasion of colon cancer cells from oncogenic stimulation by EGF (14). Hence, we believe that UVC could be applied for the clinical strategy against human malignancies including pancreatic cancer. We herein investigated the effect of UVC on pancreatic cancer cell migration and showed that UVC strongly suppressed PDGF-BB-induced migration in AsPC1 and BxPC3 cells.

## Materials and methods

### Materials

Recombinant human PDGF-BB was purchased from R&D Systems, Inc. (Minneapolis, MN). The anti-GAPDH antibody was purchased from Santa Cruz Biotechnology, Inc. (Santa Cruz, CA). Antibodies against phospho-p44/p42 mitogen-activated protein kinase (MAPK), phospho-p38 MAPK, phospho-stress-activated protein kinase/c-Jun-N-terminal kinase (SAPK/JNK), phospho-Akt and phospho-GSK-3β were purchased from Cell Signaling, Inc. (Beverly, MA). The ECL Western blot detection system was purchased from GE Healthcare (Buckinghamshire, UK). Other materials and chemicals were obtained from commercial sources.

### Cell culture

AsPC1 and BxPC3 pancreatic cancer cells were grown in Roswell Park Memorial Institute RPMI-1640 (Invitrogen, San Diego, CA) supplemented with 10% heat-inactivated fetal calf serum (FCS), penicillin (100 U/ml) and streptomycin (100 μg/ml) (all from Invitrogen), in a humidified 5% CO_2_ incubator at 37°C. Unless indicated otherwise, they were incubated in serum-free medium for 24 h before experiments as previously described (13).

### Cell migration assay

Cell migration was assessed using a Boyden chamber (8 μm pores, Transwell^®^; Corning Costar Corp., Cambridge, MA). The cells (AsPC1 and BxPC3) were exposed to UVC (0–100 J) and incubated for 6 h. These cells (5×10^4^ per well) were then seeded onto the upper chamber in RPMI containing 10% FCS. After a 16-h incubation at 37°C, the cells were treated with PDGF-BB at the indicated concentrations for 36 h. The cells were then fixed and stained with 1 ml of clonogenic reagent (50% ethanol, 0.25% 1,9-dimethyl-methylene blue) for 30 min. The cells on the upper surface of the membrane were then mechanically removed, and the cells that had migrated to the lower surface of the membrane were observed. The average number of migrated cells from 5 randomly chosen fields on the lower surface of the membrane was counted. All data were obtained from at least three independent experiments.

### Western blot analysis

Western blot analyses were performed as previously described (15). In brief, the protein lysates (5 μg) were fractionated and transferred onto an Immune-Blot PVDF Membrane (Bio-Rad Laboratories, Hercules, CA). Membranes were blocked with 5% fat-free dry milk in phosphate-buffered saline containing 0.1% Tween-20 for 30 min before incubation with the indicated primary antibodies. Peroxidase-labeled antibodies were used as secondary antibodies. The peroxidase activity on the membrane was visualized on X-ray film by means of the ECL Western blot detection system.

### Densitometric analysis

The densitometric analysis was performed using a scanner and an image analysis software package (Image J ver. 1.32). The background-subtracted signal intensity of each protein signal was normalized to the respective control signal. All data were obtained from at least three independent experiments.

### Statistical analysis

The data were analyzed by ANOVA followed by the Bonferroni method for multiple comparisons between the indicated pairs, and p<0.05 was considered to be significant.

## Results

### PDGF-BB causes migration in AsPC1 and BxPC3 pancreatic cancer cells

**Figure 1:**
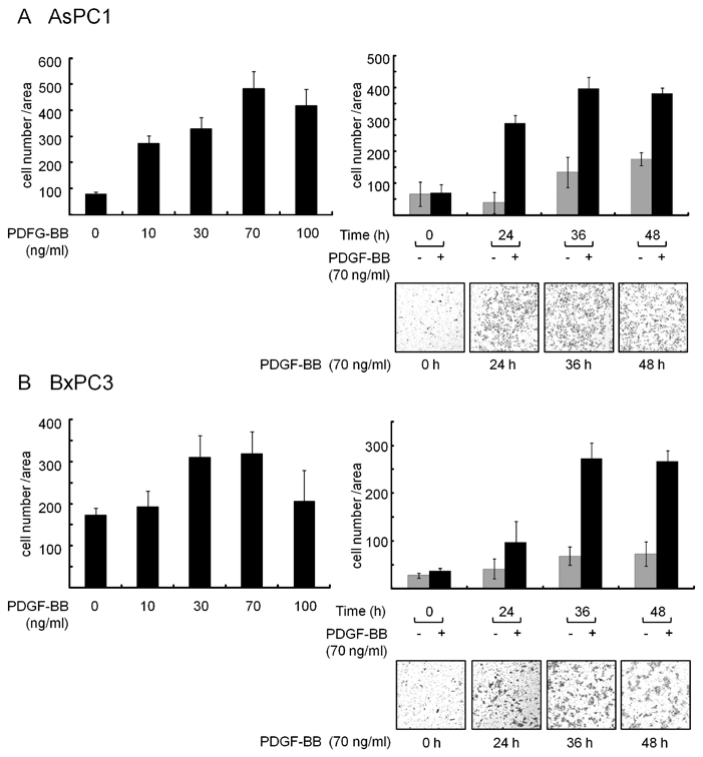
The effects of PDGF-BB on the migration of AsPC1 and BxPC3 pancreatic cancer cells. AsPC1 (A) and BxPC3 (B) cells (5×10^4^ per well) were seeded in the upper Boyden chamber in RPMI-1640 containing 10% fetal calf serum, and after a 16 h incubation, the cells were treated with the various concentrations of PDGF-BB (0–100 ng/ml) for the indicated periods at 37°C. The cells were then fixed and stained as described in Materials and methods. The average number of migrated cells from 5 randomly chosen fields on the lower surface of the membrane was counted. Each value represents the mean ± SEM of triplicate independent determinations. All data were obtained from at least three independent experiments. Lower panels below right graphs show the cells which were migrated to the lower surface of the membrane.

We first examined the effect of PDGF-BB on migration in AsPC1 and BxPC3 cells. After incubation of the cells (5×10^4^/well) on the upper Boyden chamber Transwell, they were treated with PDGF-BB at the indicated concentrations for 24 h. As depicted in Fig. 1, PDGF-BB increased the number of migrated cells which were observed on the lower surface of the membrane. Maximum effects on the migration were seen when they were exposed to PDGF-BB at a dose of 70 ng/ml (Fig. 1, left bar graph, respectively). In addition, time-course experiments showed that 36 h incubation with PDGF-BB exerted sufficient effects on migration of these cells (Fig. 1, right bar graph and panels, respectively). Therefore, we used 70 ng/ml of PDGF-BB and incubated for 36 h after treatment in the following experiments.

### PDGF-BB caused phosphorylation of p44/p42 MAPK, SAPK/JNK, Akt and GSK-3β, but not p38 MAPK in AsPC1 and BxPC3 cells

**Figure 2:**
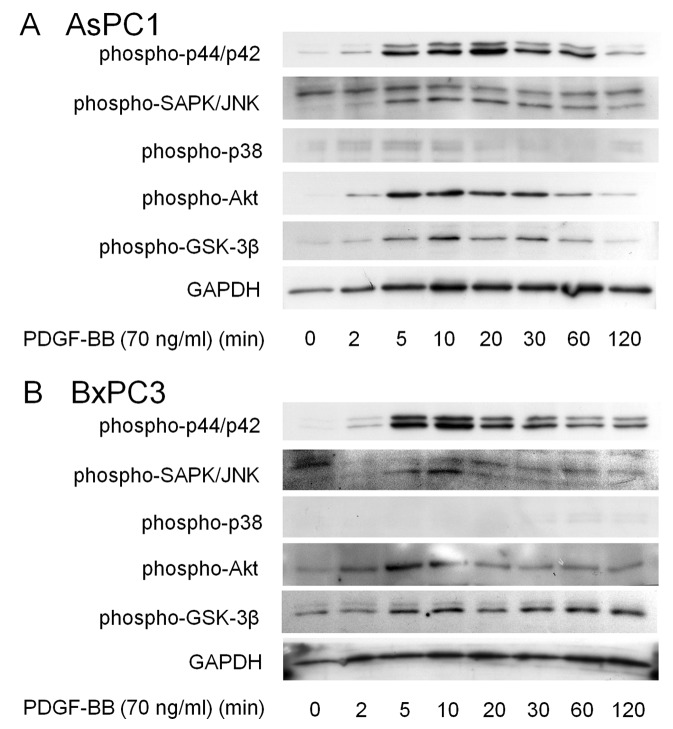
PDGF-BB induces phosphorylation of p44/p42 MAPK, SAPK/JNK, Akt and GSK-3β in AsPC1 and BxPC3 pancreatic cancer cells. AsPC1 (A) and BxPC3 (B) cells were exposed to 70 ng/ml of PDGF-BB for the indicated periods (0–120 min). The extracts of cells were then subjected to sodium dodecyl sulfate-polyacrylamide gel electrophoresis (SDS-PAGE) and were then subjected to the Western blot analysis with antibodies against phospho-specific p44/p42 MAPK, SAPK/JNK, p38 MAPK, Akt, GSK-3β and GAPDH.

In order to elucidate how PDGF-BB causes pancreatic cancer cell migration, we next examined the effects of PDGF-BB on the activation of several kinase cascades. Western blotting revealed that PDGF-BB induced activation of p44/p42 MAPK, SAPK/JNK and Akt in AsPC1 cells (Fig. 2A). GSK-3β is a critical downstream element of the PI3K/Akt cell survival pathway, and its activity can be inhibited by Akt-mediated phosphorylation (16) and we observed that PDGF-BB induced phosphorylation of GSK-3β. However, PDGF-BB had little effect on p38 MAPK in these cells (Fig. 2A). Since similar effects were also observed in BxPC3 cells (Fig. 2B), these results led us to further investigate which kinase plays a critical role in migration induced by PDGF-BB in pancreatic cancer cells.

### Pretreatement with UVC suppresses PDGF-BB-induced migration in AsPC1 and BxPC3 cells

**Figure 3:**
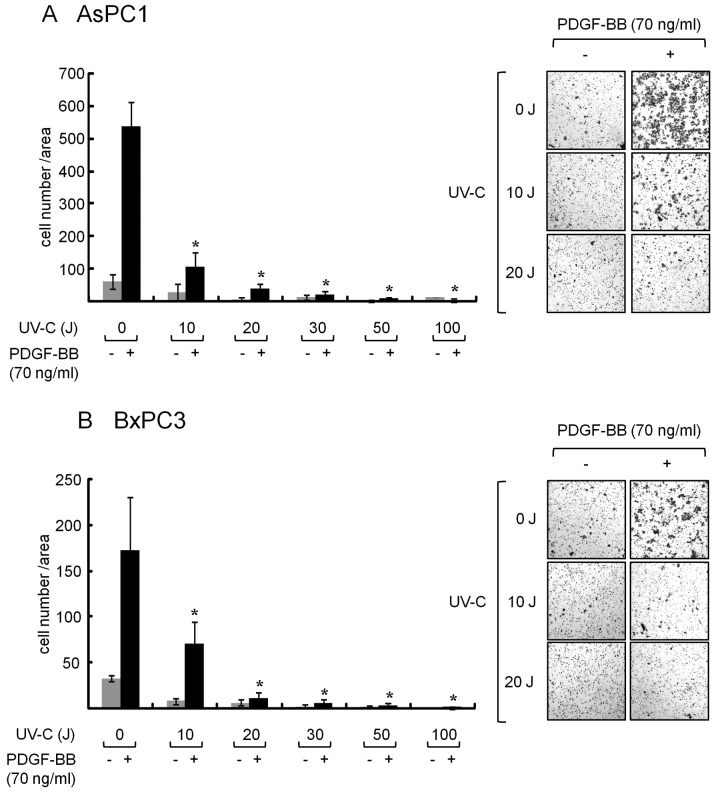
The effect of UVC on PDGF-BB-induced migration of AsPC1 and BxPC3 pancreatic cancer cells. AsPC1 (A) and BxPC3 (B) cells were first exposed to UVC at the indicated doses (0–100 J) and incubated for 6 h. These cells (5×10^4^ per well) were then seeded in the upper Boyden chamber in RPMI-1640 containing 10% fetal calf serum, and after a 16 h incubation, the cells were incubated with 70 ng/ml of PDGF-BB for 36 h at 37°C. The cells were then fixed and stained as described in Materials and methods. The average number of migrated cells from 5 randomly chosen fields on the lower surface of the membrane was counted. Each value represents the mean ± SEM of triplicate independent determinations. All data were obtained from at least three independent experiments. Right panels shows the cells which migrated to the lower surface of the membrane under the indicated conditions. ^*^Significant increase (p<0.01) compared with the control (lane 2, respectively).

We previously reported that UVC induced downregulation of the EGFR, which leads to cell growth inhibition in pancreatic cancer cells (12). Moreover, our recent study showed that UVC can induce apoptosis in pancreatic cancer cells via the mitochondrial pathway (13). Therefore, we next examined whether UVC affects migration induced by PDGF-BB in AsPC1 and BxPC3 cells. As shown in Fig. 3, when the cells were pretreated with increasing doses of UVC and then exposed to PDGF-BB, the number of migrated cells was clearly decreased (Fig. 3). The inhibitory effect of UVC on migration was seen at a dose over 10 J in these cells. These results strongly suggest that UVC has a suppressive effect on pancreatic cancer cell migration.

**Figure 4:**
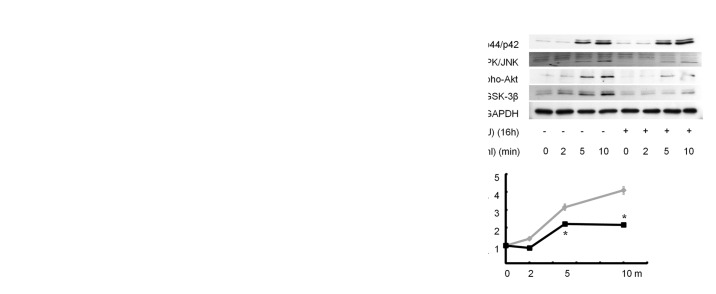
UVC suppresses PDGF-BB-induced phosphorylation of Akt and GSK-3β, but not p44/p42 MAPK and SAPK/JNK in AsPC1 and BxPC3 pancreatic cancer cells. (A) AsPC1 and (B) BxPC3 cells were exposed to 10 J of UVC and incubated for 16 h. They were then exposed to 70 ng/ml of PDGF-BB for the indicated periods (0–10 min). The extracts of cells were subjected to sodium dodecyl sulfate-polyacrylamide gel electrophoresis (SDS-PAGE) and were then subjected to the Western blot analysis with antibodies against phospho-specific p44/p42 MAPK, SAPK/JNK, Akt, GSK-3β and GAPDH. The lower graph shows the quantification data for the relative levels of phospho-Akt, after normalization with respect to GAPDH, as determined by densitometry. Each value represents the mean ± SEM of triplicate independent determinations. ^*^Significant decrease (p<0.05) compared with the corresponding control (no treatment with UVC).

Pretreatment with UVC suppressed PDGF-BB-induced phosphorylation of Akt and GSK-3β in AsPC1 and BxPC3 cells. In order to elucidate how UVC suppressed PDGF-BB-induced migration in pancreatic cancer cells, we next examined the effect of UVC on several kinase cascade induced by PDGF-BB. While PDGF-BB activated p44/p42 MAPK, SAPK/JNK, Akt and GSK-3β, but not p38 MAPK (Fig. 2), pretreatment with UVC significantly inhibited PDGF-BB-induced phosphorylation of Akt and GSK-3β in these cells. Since UVC failed to affect p44/p42 MAPK and SAPK/JNK by PDGF-BB (Fig. 4), it is likely that UVC inhibits PDGF-BB-induced migration by suppressing Akt-GSK-3β pathway in pancreatic cancer cells.

## Discussion

**Figure 5:**
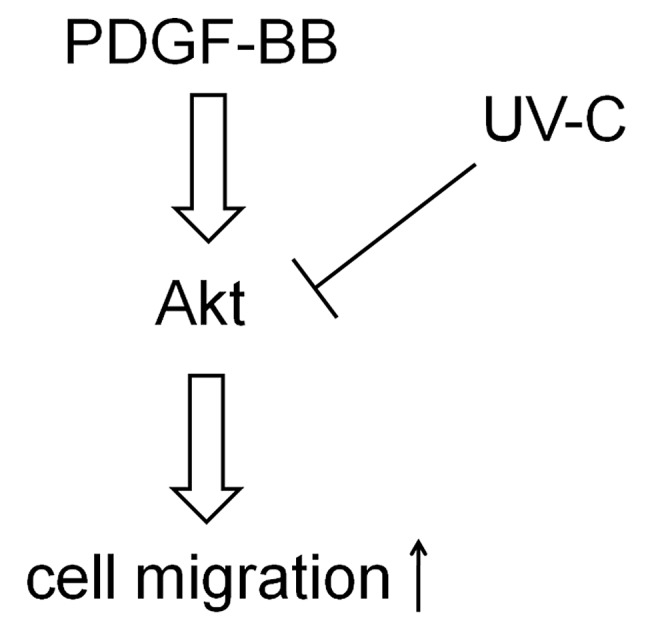
Hypothetical mechanism underlying the suppressive effect of UVC on pancreatic cancer cell migration. In pancreatic cancer cells, PDGF-BB strongly induces cell migration via the Akt pathway. However, UVC suppresses phosphorylation of Akt and subsequent GSK-3β, resulting in the inhibition of pancreatic cancer cell migration.

We recently demonstrated the potential applicability of UVC treatment in patients with pancreatic cancer (12,13). In these studies, we showed that UVC causes cell growth inhibition as well as apoptosis in human pancreatic cancer cells. In our present study, we provide novel evidence that UVC inhibits cancer cell migration by suppressing the Akt-GSK-3β pathway. Using the migration assay, we first showed that PDGF-BB, which is known to play a critical role in invasion and metastasis in human cancers (7,8), caused migration in AsPC1 and BxPC3 cells (Fig. 1). Moreover, pretreatment with UVC significantly inhibited PDGF-BB-induced migration of these cells (Fig. 3). Since PDGF-BB induced phosphorylation of p44/p42 MAPK, SAPK/JNK, Akt and GSK-3β in these cells (Fig. 2), we next examined the involvement of these kinases in PDGF-BB-induced cell migration. Interestingly, UVC suppressed PDGF-BB-induced phosphorylation of Akt and subsequent GSK3β, but not p44/p42 MAPK and SAPK/JNK (Fig. 4). Therefore, our results suggest that UVC inhibits PDGF-BB-induced migration by suppressing the Akt-GSK-3β pathway in pancreatic cancer cells. A schematic representation of the hypothetical mechanism by which UVC inhibits cell migration is shown in Fig. 5.

We previously reported that Rho-kinase, which is a downstream kinase of Rho, negatively regulates the migration of colon cancer cells (17). In this study, Rho-kinase inhibitor induced colon cancer cell migration by disrupting focal adhesion formation via the Akt pathway. In the present study, we showed that PDGF-BB-induced migration was mediated through activation of Akt and that UVC inhibited cell migration by suppressing the Akt pathway (Fig. 5). Our present findings could allow us to consider the possibility of UVC as a therapeutic strategy for pancreatic cancer, although further investigation is required on the detailed mechanism of UVC suppression of the Akt pathway. In addition, the development of devices that supply UVC radiation efficiently is also required for future clinical application. For example, delivery of UVC radiation to the pancreas through endoscopic retrograde assisted cholangiopancreatography could be attained. Moreover, accumulating evidence shows that extracorporeal photochemotherapy, or photopheresis, which is a low-risk therapeutical intervention, has been applied to a variety of hematological malignancies, autoimmune conditions and transplantation (18). As for cancer therapy, the mode of action of photopheresis encompasses apoptosis-induction and modifications of immunoregulatory processes, leading to the elimination of malignant cells, as well as the down-modulation of harmful immune responses (18). Therefore, the therapeutic action of extracorporeal UV irradiation of circulating blood, where metastatic cells exist, would be worthwhile to evaluate the possibility of its application to patients with advanced cancer. In conclusion, our results suggest that UVC irradiation suppresses PDGF-BB-induced migration via the Akt pathway in human pancreatic cancer cells.

## References

[1] Parkin DM, Bray F, Ferlay J, Pisani P (2005). Global cancer statistics, 2002. CA Cancer J Clin.

[2] Riento K, Ridley AJ (2003). Rocks: multifunctional kinases in cell behaviour. Nat Rev Mol Cell Biol.

[3] Burridge K (1981). Are stress fibres contractile?. Nature.

[4] Humphries JD, Wang P, Streuli C, Geiger B, Humphries MJ, Ballestrem C (2007). Vinculin controls focal adhesion formation by direct interactions with talin and actin. J Cell Biol.

[5] Burridge K, Chrzanowska-Wodnicka M (1996). Focal adhesions, contractility, and signaling. Annu Rev Cell Dev Biol.

[6] Wang Z, Ahmad A, Li Y (2010). Emerging roles of PDGF-D signaling pathway in tumor development and progression. Biochim Biophys Acta.

[7] Henriksen R, Funa K, Wilander E, Backstrom T, Ridderheim M, Oberg K (1993). Expression and prognostic significance of platelet-derived growth factor and its receptors in epithelial ovarian neoplasms. Cancer Res.

[8] Uren A, Merchant MS, Sun CJ (2003). Beta-platelet-derived growth factor receptor mediates motility and growth of Ewing’s sarcoma cells. Oncogene.

[9] Latonen L, Laiho M (2005). Cellular UV damage responses-functions of tumor suppressor p53. Biochim Biophys Acta.

[10] Olsen BB, Neves-Petersen MT, Klitgaard S, Issinger OG, Petersen SB (2007). UV light blocks EGFR signalling in human cancer cell lines. Int J Oncol.

[11] Kim SC, Park SS, Lee YJ (2008). Effect of UV irradiation on colorectal cancer cells with acquired TRAIL resistance. J Cell Biochem.

[12] Yamauchi T, Adachi S, Yasuda I (2011). UV-C irradiation induces downregulation of EGF receptor via phosphorylation at serine 1046/1047 in human pancreatic cancer cells. Radiat Res.

[13] Yamauchi T, Adachi S, Yasuda I (2011). Ultra-violet irradiation induces apoptosis via mitochondrial pathway in pancreatic cancer cells. Int J Oncol.

[14] Adachi S, Yasuda I, Nakashima M (2011). Ultraviolet irradiation can induce evasion of colon cancer cells from stimulation of epidermal growth factor. J Biol Chem.

[15] Adachi S, Nagao T, Ingolfsson HI (2007). The inhibitory effect of (−)-epigallocatechin gallate on activation of the epidermal growth factor receptor is associated with altered lipid order in HT29 colon cancer cells. Cancer Res.

[16] Cross DA, Alessi DR, Cohen P, Andjelkovich M, Hemmings BA (1995). Inhibition of glycogen synthase kinase-3 by insulin mediated by protein kinase B. Nature.

[17] Adachi S, Yasuda I, Nakashima M (2011). Rho-kinase inhibitor upregulates migration by altering focal adhesion formation via the Akt pathway in colon cancer cells. Eur J Pharmacol.

[18] Szodoray P, Papp G, Nakken B, Harangi M, Zeher M (2010). The molecular and clinical rationale of extracorporeal photochemotherapy in autoimmune diseases, malignancies and transplantation. Autoimmun Rev.

